# Inhibition of Na^+^, K^+^ -ATPase with ouabain is detrimental to equine blastocysts

**DOI:** 10.21451/1984-3143-AR2019-0079

**Published:** 2020-01-22

**Authors:** Agnelo Douglas do Nascimento, Juliana Carla Cavalcanti Marques, Allan Rodolf Ribeiro Cezar, André Mariano Batista, John Patrick Kastelic, Diogo Ribeiro Câmara

**Affiliations:** 1 Departamento de Medicina Veterinária, Laboratório de Reprodução Animal, Universidade Federal de Alagoas, Viçosa, AL, Brasil; 2 Departamento de Medicina Veterinária, Universidade Federal Rural de Pernambuco, Recife, PE, Brasil; 3 Department of Production Animal Health, Faculty of Veterinary Medicine, University of Calgary, Calgary, AB, Canada

**Keywords:** embryology, horse embryo, sodium pump

## Abstract

Although equine blastocysts ≤ 300 µm in diameter can be successfully vitrified, larger equine blastocysts are not good candidates for cryopreservation. As Na^+^, K^+^-ATPase is involved in maintaining blastocyst expansion, perhaps inhibition of this enzyme would be a viable method of reducing blastocyst diameter prior to cryopreservation. Objectives were to evaluate effects of ouabain-induced inhibition of Na^+^, K^+^-ATPase in equine blastocysts. Sixteen mares were ultrasonographically monitored, given deslorelin acetate to induce ovulation, and inseminated. Embryos (D7 and D9) were harvested and Na^+^, K^+^-ATPase inhibited for 1 or 6 h by exposure to 10^-6^ M ouabain, either natural ouabain or conjugated to fluorescein (OuabainFL), during incubation at 37° C. Evaluations included morphometric characteristics (bright field microscopy) and viability (Hoescht 33342 + propidium iodide). Blastocysts incubated for 6 h in Holding medium + ouabain (n=3) had, on average, a 45.7% reduction in diameter, with adverse morphologic features and no re-expansion after subsequent incubation in Holding medium for 12 h. In subsequent studies, even a 1-h exposure to Ouabain or OuabainFL, caused similar reductions, namely 38.7 ± 6.7% (n=5) and 33.6 ± 3.3% (n=7) for D7 and D9 blastocysts, respectively. Ouabain binding was confirmed after OuabainFL exposition and all embryos (n=12) lost viability. We concluded that Na^+^, K^+^-ATPase inhibition with ouabain caused death of equine blastocysts and therefore was not a viable method of reducing blastocyst size prior to cryopreservation.

## Introduction

Cryopreservation of equine embryos has been a challenge due to capsule development, mitotic activity and in particular, embryo size (Legrand et al., 2001; Stout, 2012). Vitrification has become the preferred method to cryopreserve embryos of many species. Furthermore, there are various strategies to combine, add and remove cryoprotectants, as well as various processing techniques (Vajta and Gjerris, 2006).

The first foal born after vitrification was reported by Yamamoto et al. (1982), with only 9% of frozen-thawed blastocysts resulting in live foals. Currently, cryopreservation of equine embryos ≤ 300 µm of diameter resulted in satisfactory pregnancy rates (64 to 80%; Hochi et al., 1996). However, pregnancy rates decreased when horse embryos > 300 µm were vitrified (MacLellan et al., 2001). When equine embryos were biopsied with a micromanipulator, they collapsed, but the embryos remained viable (Choi et al., 2010). The same authors subsequently evaluated effects of blastocoel cavity collapse in large embryos (330 to 730 μm) before vitrification and achieved pregnancy rates of approximately 50% (Choi et al., 2011). However, equipment required for micromanipulation is expensive. Therefore, perhaps establishment of chemically defined medium to reduce horse blastocyst diameter could promote use of vitrification of large equine embryos in the field. Strategies that interfere with cellular cytoskeleton, such as cytochalasin-B, resulted in higher rates of porcine embryo survival post-thawing (Dobrinsky et al., 2000), but no positive influence on thawed equine embryos was reported, although authors stated that reduction of the water ratio in expanded blastocysts could improve effectiveness of cryopreservation (MacLellan et al., 2001).

Pre-implantation embryo development is influenced by Na^+^, K^+^-ATPase activity, promoting embryo cavitation and modifying ionic gradients on trophectoderm epithelium, interfering with formation, distribution, and permeability of tight junctions (TJ) between trophoblastic cells (Manejwala et al., 1989; Budik et al., 2008; Giannatselis et al., 2011). Therefore, the objective was to evaluate effects of Na^+^, K^+^-ATPase inhibition on morphological characteristics and viability of equine blastocysts, with a long-term goal of reducing diameter to promote vitrification.

## Methods

This study was approved by the Ethics Committee for Animal Experimentation of the Federal University of Alagoas (protocol number 35/2017). Sixteen mares and one Margalarga Marchador stallion, located in Viçosa-AL, Brazil (9°23' S; 36°15' O) were used. These horses were raised in semi-extensive conditions, with free access to good quality water and grass, plus supplementation with 4 kg concentrate/head/d. All horses were deemed breeding sound and there was no indication of any reproductive disorder. Ovarian follicular growth and uterine characteristics of mares were monitored every 2 d, with transrectal ultrasonography. Mares in estrus with follicles > 35 mm and uterine edema grade 3 (0-5; Samper, 1997) were given deslorelin acetate (1 mg/IV), to induce ovulation.

Twenty-four hours after deslorelin injection, if ovulation was confirmed by ultrasonography, the mares were artificially inseminated with 5 × 10^8^ motile sperm, using routine procedures (Aurich et al., 1997) on D0. On D7 or D9, the mare’s uterus was flushed three times (Ringer's solution) to collect embryos, using a closed system. The remaining fluid in the filter was searched in a sterile Petri dish and recovered embryos were washed 10 times in Holding solution (EquiHold, Minitube, Tienfenbach, Germany), loaded into 0.5 mL straws and further transported to the laboratory, within 30 min.

Immediately after arriving at the laboratory, embryos were transferred to a new Petri dish containing Holding medium (100 µl) covered by sterile mineral oil (200 µl), and kept for 1 h at 37 °C for equilibration. Thereafter, embryos were morphologically evaluated (McKinnon and Squires, 1988) using an inverted microscope (Medilux MDL-INV-1, Biosystems). Furthermore, digital images were captured and embryo diameters determined with a micrometric ruler (1 mm) and ImageJ software (Version 1.52a, Schneider et al., 2012). Diameter reduction of each embryo was calculated as a percentage, based on embryo diameter after 1 h incubation in Holding medium (37 ° C), considered as 100% of initial diameter, and after ouabain exposure, as follows (Equation 1):

$$\text{Diameter reduction}\left(\text{\%}\right)\text{=}\frac{\begin{array}{c} \text{100 × final embryo diameter}\\ \left(\text{after ouabain exposure}\right) \end{array}}{\begin{array}{c} \text{Initial embryo diameter}\\ \left(\text{before ouabain exposure}\right) \end{array}}$$ (1)

In total, 17 embryos were recovered, all classified as Grade I. Two blastocysts D7 were incubated only in Holding medium for 24 h at 37 °C to evaluate the influence of culture system on embryo quality. Two D7 and one D9 blastocysts, after initial incubation in Holding medium (1 h), were transferred and maintained for 6 h in Holding medium + Na^+^, K^+^-ATPase specific inhibitor (ouabain, 10^-6^ M; Sigma-Aldrich, São Paulo, SP, Brazil), under the same initial incubation conditions. Thereafter, blastocysts were withdrawn from ouabain-treated medium and incubated for 12 h in Holding media, to assess embryo re-expansion. During this interval, blastocysts were photomicrographed within 1 h and subsequent morphological and morphometric evaluations performed.

Based on results of the first experiment, it was decided to evaluate shorter intervals of Na^+^, K^+^-ATPase inhibition and assess embryos using fluorescent probes. Propidium iodide (PI, 10 μg/mL, Sigma-Aldrich), Hoechst 33342 (10 μg/mL, Sigma-Aldrich), fluorescent-conjugated ouabain (10^-6^ M, Bobipy™ FL ouabain; Invitrogen, São Paulo, SP, Brazil) and ouabain (10^-6^ M) were used after dilution in Holding medium. Analyses were performed using epifluorescence microscopy (Feldmann Wild Leitz- 3500T FL), with excitation/emission filters of 533/617, 361/497, 503/512 for PI, Hoechst 33342 and Bobipy™ FL ouabain (OuabainFL), respectively.

Six D9 blastocysts and one D7 were incubated (1 h, 37 °C) in Holding + ouabain, followed by Hoescht and PI staining to evaluate membrane integrity of trophoblastic cells. Moreover, to confirm ouabain-embryo binding, four D7 and one D9 blastocysts were incubated (1 h, 37 °C) in Holding + OuabainFL + Hoechst, followed by IP staining. Before and after incubation, blastocysts were photomicrographed in bright field, to assess morphology and diameter.

Results of the present study are predominantly presented using descriptive statistics. Average diameter reduction (%) of D7 and D9 blastocysts exposed to either ouabain or ouabainFL were compared after normality analysis (Shapiro-Wilk test), followed by Student’s *t*-test, with data presented as means ± SEM. Results were considered significant when P<0.05.

## Results

**Figure 1:**
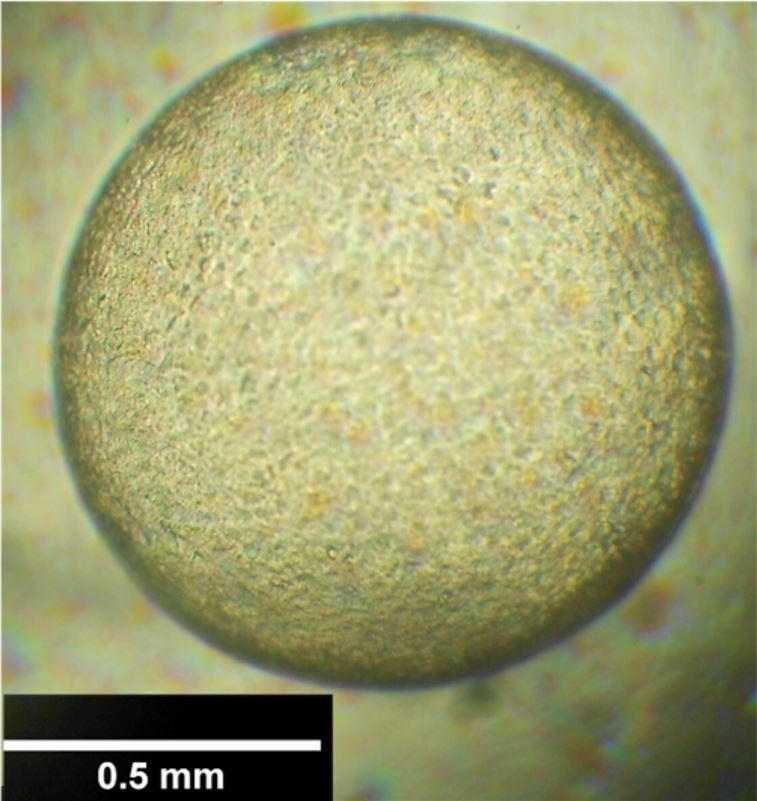
Equine blastocyst recovered at D7, after 24 h in Holding medium at 37 °C, with morphologic characteristics compatible with Grade 1 (1-5).

**Figure 2:**
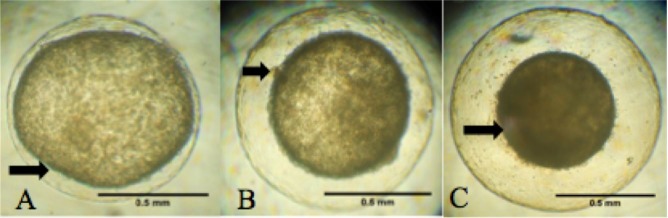
Equine blastocyst recovered at D9, after 1 h exposed to Holding + ouabain (10^-6^ M, (A)); after ouabain exposure, withdrawn and maintained for 3 h (B) or 6 h (C) in Holding medium. Note morphological alterations in the embryo (arrows), e.g. capsule detachment (A), granular surface and trophoblastic cells of various sizes (B) and culminating with blastocoel collapse (C).

Both D7 blastocysts kept in Holding medium preserved their morphologic features and increased in diameter during 24 h incubation (Figure 1). The three blastocysts maintained in Holding + ouabain (10^-6^ M) during 6 h reduced their diameter 35.9, 48.7 and 52.5% (D7, D7 and D9, respectively), with no subsequent re-expansion after up to 12 h in Holding medium (Figure 2 – A to C).

**Figure 3:**
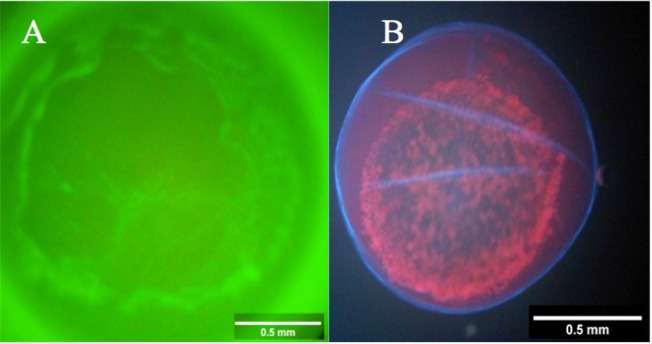
Equine embryo (D9) exposed to ouabain conjugated to fluorescent probe (A) indicating ouabain binding to Na^+^, K^+^-ATPase. Equine embryo (D9), after incubation (1 h), in 10^-6^ M ouabain in Holding medium + followed by exposure to Hoechst 33342 and propidium iodide, indicating loss of viability (B).

Regarding fluorescent analysis, it was observed that ouabain bound to trophoblastic cells (Figure 3A), confirming the presence of Na^+^, K^+^-ATPase. Moreover, all (12/12) of blastocysts exposed to ouabain or ouabainFL were positive for PI staining after incubation (Figure 3B). It was noteworthy that blastocysts D7 and D9 had similar diameter reductions after 1 h incubation, regardless of exposure to ouabain or ouabainFL. Blastocysts D7 (n=5) reduced 38.7 ± 6.7% and D9 (n=7) reduced 33.6 ± 3.3% (P>0.05). Furthermore, all blastocysts had adverse morphologic features, including heterogeneous texture (12/12), extrusion (12/12), capsule detachment (10/12) and irregular shape (5/12).

Two D7 blastocysts were removed from Holding + ouabain medium after 1 h of incubation and transferred to Holding medium for up to 6 h, but no further re-expansion was observed, confirming a lack of viability.

## Discussion

To our knowledge, this was the first study that primarily aimed to evaluate effects of Na^+^, K^+^-ATPase inhibition in equine embryos. Although embryos were not incubated in a CO_2_ atmosphere, morphologic characteristics of blastocysts maintained in Holding medium for 24 h indicated that our incubation system sustained embryo viability. Similarly, equine embryos cultured in MEM at 37 °C for 12 h also had normal development (Clark et al., 1987)

Exposure of 3 blastocysts to ouabain for 6 h, resulting in blastocoel collapse and no subsequent re-expansion, even after 12 h incubated in Holding medium (without ouabain), indicated that Na^+^, K^+^-ATPase had a crucial role in supporting the blastocoel during this embryonic stage. Waelchli et al. (1997), evaluating trophoblastic vesicles from equine embryos, reported that 78% of vesicles collapsed after Na^+^, K^+^-ATPase inhibition with ouabain. Although Waelchli et al. (1997) focused on trophoblastic vesicles, the authors cited that D-9 horse blastocysts partially collapsed after exposure to ouabain (10^-6^ M), with further re-expansion in ouabain-free medium in a pilot study, although that was not observed in the present study.

We demonstrated that ouabain, a specific Na^+^, K^+^-ATPase inhibitor, reduced diameter of D7 and D9 equine blastocysts, presumably by interfering with fluid exchange through the trophectoderm, similar to a previous report with D-9 equine embryos (Waelchli et al., 1997). It is noteworthy that Na^+^, K^+^-ATPase was detected in embryos of several species. Furthermore, there were several isoforms, depending on composition of specific α (α1, α2, and α3) or β (β1, β2, and β3) subunits that comprise the protein (Câmara et al., 2017). Although Budik et al. (2008) reported an absence or minimal expression of α1 and β1 subunits of Na^+^, K^+^-ATPase in D8 equine embryos and a remarkable increase in D10, the present study demonstrated that equine embryos were influenced by Na^+^, K^+^-ATPase inhibition as early as D7. As ouabain concentration used in the present study (10^-6^ M) was expected to inhibit the α1 subunit, which comprises Na^+^, K^+^-ATPase isoform less sensitive to ouabain (Blanco, 2005), it is feasible that other isoforms could be also inhibited, as Betts et al. (1997) demonstrated that bovine embryos express α3 subunit of Na^+^, K^+^-ATPase as early as the morula stage.

The use of OuabainFL confirmed that ouabain bound to equine blastocysts. In spite of ouabain type (ouabain or ouabainFL) and briefer exposure to ouabain (1 h), and embryonic stage (D7 or D9 blastocyst), there was a reduction on blastocyst diameter and adverse effects on morphology after Na^+^, K^+^-ATPase inhibition. Furthermore, damage to trophoblastic membranes was demonstrated, as PI is capable of crossing only a damaged cell membrane (Van Engeland et al., 1998; Willingham, 1999), reinforcing the negative morphologic aspects of the blastocysts.

Blastocysts expand due to continuous fluid movement through trophectoderm, although that does not happen until tight junctions (TJ) are fully developed and functional between trophectoderm cells, maintaining blastocoel fluid and epithelial integrity (Watson and Barcroft, 2001). In previous studies, inhibition of Na^+^, K^+^-ATPase resulted in increased intracellular Na^+^ concentrations, inhibition of Na^+^, Ca^2+^ ionic channels and an elevation of intracellular Ca^2+^ and AMPc, compromising E-cadherin/catenin system, leading to TJ disruption and impairment of cell membrane polarization control (Eckert and Fleming, 2008; Rajasekaran et al., 2001). We inferred that functional inhibition of Na^+^, K^+^-ATPase interfered with formation/function of TJ on trophoblastic cells (manifested by ability of PI to cross the membrane) as well as induced trophectoderm membrane damage.

## Conclusions

Inhibition of Na^+^, K^+^-ATPase with ouabain caused embryonic death and therefore, this approach is not a viable option for chemical reduction of equine blastocyst size prior to vitrification. Future studies, at molecular levels, should be undertaken to evaluate effects of Na^+^, K^+^-ATPase inhibition on tight junction structure and function in equine embryos.
